# Association between ambient air pollutants and short-term mortality risks during 2015–2019 in Guangzhou, China

**DOI:** 10.3389/fpubh.2024.1359567

**Published:** 2024-03-04

**Authors:** Yuyang Chen, Sili Chen, Lei Zhang, Weishan Kang, Guozhen Lin, Qiaoyuan Yang

**Affiliations:** ^1^School of Anesthesiology, Southern Medical University, Guangzhou, China; ^2^Department of Preventive Medicine, School of Public Health, Guangzhou Medical University, Guangzhou, China; ^3^Guangzhou Center for Disease Control and Prevention, Guangzhou, China; ^4^Guangdong Provincial Key Laboratory of Major Obstetric Diseases, Guangdong Provincial Clinical Research Center for Obstetrics and Gynecology, The Third Affiliated Hospital of Guangzhou Medical University, Guangzhou, China

**Keywords:** ambient air pollutants, all-cause mortality, short-term, time-series study, air pollution

## Abstract

With the development of technology and industry, the problem of global air pollution has become difficult to ignore. We investigated the association between air pollutant concentrations and daily all-cause mortality and stratified the analysis by sex, age, and season. Data for six air pollutants [fine particulate matter (PM_2.5_), inhalable particles (PM_10_), nitric dioxide (NO_2_), sulfur dioxide (SO_2_), ozone (O_3_), and carbon monoxide (CO)] and daily mortality rates were collected from 2015 to 2019 in Guangzhou, China. A time-series study using a quasi-Poisson generalized additive model was used to examine the relationships between environmental pollutant concentrations and mortality. Mortality data for 296,939 individuals were included in the analysis. The results showed that an increase of 10 μg/m^3^ in the concentrations of PM_2.5_, PM_10_, SO_2_, O_3_, NO_2_, and CO corresponded to 0.84% [95% confidence interval (CI): 0.47, 1.21%], 0.70% (0.44, 0.96%), 3.59% (1.77, 5.43%), 0.21% (0.05, 0.36%), 1.06% (0.70, 1.41%), and 0.05% (0.02, 0.09%), respectively. The effects of the six air pollutants were more significant for male individuals than female individuals, the cool season than the warm season, and people 75 years or older than those younger than 75 years. PM_2.5_, PM_10_, SO_2_, and NO_2_ were all associated with neoplasms and circulatory and respiratory diseases. The two-pollutant models found that PM_2.5_, PM_10_, and NO_2_ may independently affect the risk of mortality. The results showed that exposure to PM_2.5_, PM_10_ and NO_2_ may increase the risk of daily all-cause excessive mortality in Guangzhou.

## 1 Introduction

With the rapid development of the global economy and technology, ambient air pollution has become a serious ecological problem. In 2016, it was reported that air pollution caused more than 7 million deaths worldwide (1). According to the Global Burden of Disease study, air pollution is one of the leading causes of the global burden of disease (2). This burden is especially obvious in developing countries (3). As one of the largest developing countries, China has a major problem with air pollution. In 2016, air pollution caused an estimated 1.58 million deaths in China (4).

Considerable evidence for the relationship between pollutants and mortality risk has been generated in recent years (5). Outdoor air pollution has been recognized as an important factor for many causes of death such as neoplasms, chronic respiratory diseases, coronary heart disease, and cardiovascular diseases (6–8). Fine particulate matter (PM_2.5_), inhalable particles (PM_10_), and nitrogen dioxide (NO_2_) are associated with all-cause mortality, and PM_2.5_ is also associated with increased mortality from cardiovascular disease and coronary heart disease (9, 10). Relevant research has revealed the relationship between air pollution and human lung function. Sulfur dioxide (SO_2_), NO_2_, and ozone (O_3_) exposure has been shown to reduce adult lung function and have a negative effect on health (11). Furthermore, a previous study demonstrated that PM_2.5_ and PM_10_ concentrations in Chongqing and PM_2.5_, PM_10_, and SO_2_ concentrations in Guangzhou are positively associated with lung cancer mortality (12). Other studies have shown that the concentrations of PM_2.5_, PM_10_, SO_2_, O_3_, NO_2_, and carbon monoxide (CO) are negatively correlated with the probability of biochemical pregnancy and clinical pregnancy, with the concentration of CO leading to the greatest reduction in odds (13). In another study, the concentrations of CO and NO_2_ were positively correlated with the incidence of tuberculosis in Shanghai (14). However, many studies have included only one or a few air pollutants. We analyzed the relationships between six atmospheric environmental pollutants and the number of all-cause deaths, which provides a more comprehensive and representative analysis than provided by other studies.

We used a quasi-Poisson generalized additive model (GAM) of time-series data to investigate the relationships between ambient air pollutant concentrations and daily all-cause mortality (15). This model is consistent with the recommendations of WS/T 666-2019, the National Health Commission of the People’s Republic of China. Time-series analysis is also a frequently used approach to analyze the acute health effects of daily air pollutants based on the aggregate daily date, as it can be controlled by both time-invariant and time-varying confounders. The mortality counts were low probability events; thus, they were assumed to follow a typical over-dispersed Poisson distribution. Meteorological variables, such as temperature and relative humidity, have a potential role in the association between air pollutants and human health (16, 17). The burning of fossil fuels produces ambient air pollutants that are related to health. Moreover, an increase in the amount of greenhouse gases leads to climate change and extreme weather events, which together contribute to air pollution (18, 19). Some studies have also suggested that mortality risk from diseases, such as those of the cardiovascular system, may be related to air pollution and temperature (20). Therefore, confounding factors, such as meteorological factors, were included and adjusted for in the present study to identify more rigorous relationships between daily all-cause mortality and ambient air pollutant concentrations.

Guangzhou is an important central city and the third largest city in China. Due to the rapid development of technology and industry, the impact of air pollution on people’s lives and health cannot be underestimated. Previous studies have mostly focused on the effects of air pollutants on high-risk groups, such as children and pregnant women (21, 22). At the same time, more attention to the air pollution has been paid to the link between cardiovascular disease, respiratory diseases, and flu-like illnesses and air pollution (23–25). Studies of the association between different air pollutants and daily all-cause mortality are limited. Therefore, this study was required to investigate the association between air pollution and all-cause mortality in Guangzhou.

The aim of this study was to examine the association between air pollution and daily mortality based on a GAM with a Poisson distribution, and to explore the short-term effects of six air pollutants (PM_2.5_, PM_10_, NO_2_, SO_2_, O_3_, and CO) on mortality. Stratified analysis by sex, season, and age was also performed.

## 2 Materials and methods

### 2.1 Study area

**Figure 1:**
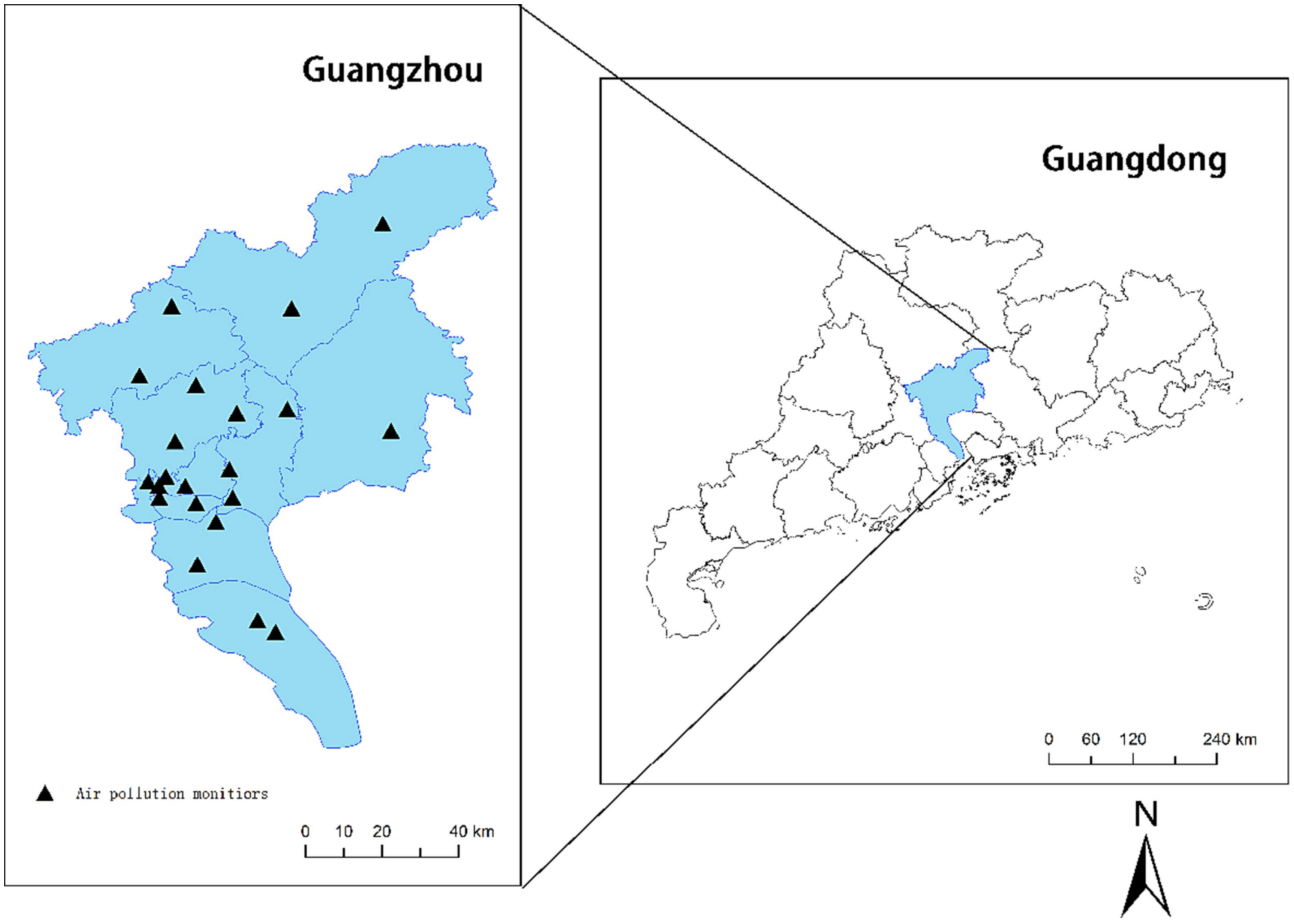
Locations of ambient air-monitoring stations in Guangzhou from 2015 to 2019.

Guangzhou, the capital city of Guangdong province, is in southern China and has a total area of 7434.40 km^2^, a permanent population of 18,734,100, and an urbanization rate of 86.48% (Figure 1). As a hilly region with a subtropical monsoon climate, Guangzhou is well-known for its mild climate and significant maritime climate characteristics. The average annual temperature in Guangzhou ranges from 21.5 to 22.2°C. It is rich in rain resources, with an average annual precipitation of more than 1,800 mm and 150 days of precipitation annually.

### 2.2 Mortality data

The daily all-cause mortality data of Guangzhou residents were obtained from the Guangzhou Center for Disease Control and Prevention from January 1, 2015 to December 31, 2019. Mortality data were collected for International Classification of Diseases, Revision 10 codes C00–D48, E00–E90, I00–I99, J00–J99, and V01–Y98, which represent neoplasms; endocrine, nutritional, and metabolic diseases; diseases of the circulatory system; diseases of the respiratory system; and external causes of morbidity and mortality, respectively.

### 2.3 Air pollution and meteorological data

During the study period, we obtained the 24-h daily average concentrations of PM_2.5_, PM_10_, SO_2_, NO_2_, and CO and the 8-h daily maximum average concentration of O_3_ from the Urban Air Quality Real-Time Release Platform^1^ of the Ministry of Ecology and Environment of the People’s Republic of China. Twenty-one air-monitoring stations in different locations throughout the city provide regular average concentrations of these pollutants (Figure 1). We also collected daily average temperature and average relative humidity data from the Guangdong Meteorological Bureau. Both parameters were incorporated into the model to improve the correction for confounding factors.

### 2.4 Statistical analyses

We employed a time-series design with a GAM to explore the short-term association between the concentrations of six ambient air pollutants and daily mortality in Guangzhou. The parameters of the GAM model and the Spearman’s correlation coefficients were obtained from a previous study (26).

Based on the results of previous time-series studies, we changed several covariates in the model to increase the reliability of our results. Degrees of freedom (df) of 5–9 years were selected based on previous studies. This main smoothing model is used for sensitivity analysis. Stratified analysis was then performed based on sex, season (warm season: May to September; cool season: October to April) and age (<64, 64–75, and ≥75 years). Additionally, we constructed two-pollutant models to determine whether the relationships were sensitive after adjustment for other gaseous air pollutants. To explain the potential delay effect, a variety of delay-effect structures was employed. All lag models fell into two classifications: single lag effects (lag0–lag5) and cumulative lag effects (lag01–lag05). Then, by adding a natural spline function with 4 df, we analyzed the exposure–response association between six ambient pollutants and mortality.

We implemented all calculations and statistical analyses with R software (version 4.1.1) using the mgcv package. All effects are expressed as excess risk (ER), calculated as (relative risk – 1) × 100%, with 95% confidence intervals (95% CIs) for children’s outpatient visits per 10-μg/m^3^ increase in ambient air pollutant concentrations. A *p-*value <0.05 was considered statistically significant.

## 3 Results

Table 1 shows the descriptive statistics of the six ambient air pollutants, daily mortality data, and meteorological conditions. A total of 296,939 deaths were recorded in Guangzhou during the study period. The daily average concentrations of PM_2.5_, PM_10_, SO_2_, O_3_, NO_2_, and CO were 34.5, 55.3, 10.4, 90.1, 46.8, and 892.8 μg/m^3^, respectively. The NO_2_ concentration was 1.2 times higher than China’s GB 3095-2012 secondary standard limit of 40 μg/m^3^ per year, and the PM_2.5_, PM_10_, and NO_2_ concentrations were 6.9, 3.7, and 4.7 times higher than the World Health Organization’s (WHO) ambient air quality standards of 5, 15, and 10 μg/m^3^ per year, respectively. The O_3_ concentration surpassed the daily standard of 160 μg/m^3^ set by China and the daily standard of 100 μg/m^3^ set by the WHO for 193 and 699 days, respectively. Disease codes C00–D48, E00–E90, I00–I99, J00–J99, and V01–Y98 accounted for 28.10, 3.66, 38.94, 14.56, and 5.31% of the total mortality, respectively. In Guangzhou, the annual mean temperature was 22.3°C, and the daily average value relative humidity was 80.3%.

**Table 1:** Daily ambient air pollution, meteorological data, and mortality rates in Guangzhou, China from January 1, 2015 to December 31, 2019.

	Mean	SD	Min	P25	P50	P75	Max
Air pollutant concentration (μg/m^3^)							
PM_2.5_	34.49	19.06	5.00	21.00	30.00	44.00	155.00
PM_10_	55.25	27.29	9.00	36.00	48.00	70.75	212.00
SO_2_	10.43	4.41	3.00	7.00	10.00	13.00	37.00
O_3_	90.10	52.31	0	49.00	84.00	122.00	287.00
NO_2_	46.85	19.12	8.00	34.00	42.50	55.00	168.00
CO	892.77	224.02	400.00	700.00	900.00	1000.00	2100.00
Meteorological measures							
Humidity (%)	80.29	10.31	31.00	75.00	82.00	88.00	100.00
Temperature (°C)	22.30	5.90	3.60	17.90	23.50	27.30	31.20
No. of daily deaths (N)	163	61	84	122	139	176	372
Sex (*N*)							
Male	92	35	40	69	79	100	220
Female	71	28	29	52	61	79	190
Age (*N*)							
<64	37	15	12	28	33	41	108
64–75	31	12	10	23	28	35	90
≥75	94	37	41	68	81	108	244
Season (*N*)							
Warm	151	59	84	116	128	148	371
Cool	171	61	86	130	148	185	372
Cause of death (*N*)							
C00–D48	46	17	17	35	40	48	118
E00–E90	6	4	0	3	5	8	28
I00–I99	63	26	20	45	55	73	164
J00–J99	24	11	6	16	21	28	70
V01–Y98	9	5	0	5	8	11	30
Other	21	10	3	15	18	25	70

The warm period was from May 1 to September 30, and the cool period was from October 1 to April 30. C00–D48, neoplasms; E00–E90, endocrine, nutritional, and metabolic diseases; I00–I99, diseases of the circulatory system; J00–J99, diseases of the respiratory system; V01–Y98, external causes of morbidity and mortality. SD, standard deviation; P25, 25th percentile; P50, 50th percentile; P75, 75th percentile.

**Figure 2:**
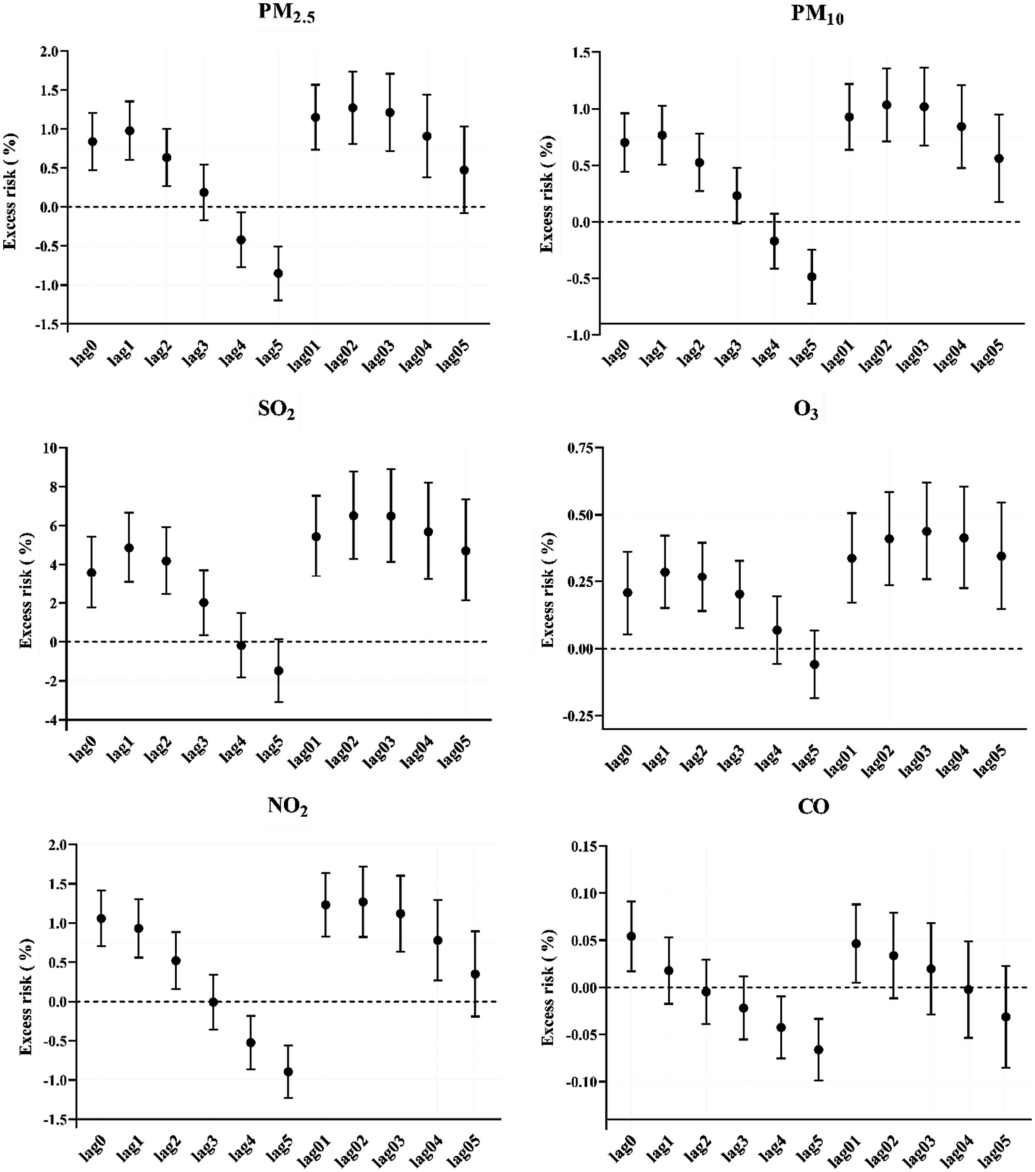
Excess risk (%) and 95% confidence intervals of mortality per 10-μg/m^3^ increase in air pollution concentration on different lag days in Guangzhou. PM_2.5_, fine particulate matter; PM_10_, inhalable particles; SO_2_, sulfur dioxide; O_3_, ozone; NO_2_, nitric dioxide; CO, carbon monoxide.

According to Figure 2, a 10-μg/m^3^ increase in PM_2.5_, PM_10_, SO_2_, O_3_, NO_2_, and CO was associated with an ER of mortality. A significant relationship between ambient air pollution concentrations and mortality was observed. In general, the effects of the six air pollutants were stronger on cumulative lag days than single lag days. Single lag days ranged from lag0 to lag5. The results showed a correlation between ambient air pollutant concentrations and mortality. Based on the model fit statistics, a 10-μg/m^3^ increase in PM_2.5_, PM_10_, SO_2_, O_3_, NO_2_, and CO on the current day corresponded to 0.84% (95% CI: 0.47, 1.21%), 0.70% (0.44, 0.96%), 3.59% (1.77, 5.43%), 0.21% (0.05, 0.36%), 1.06% (0.70, 1.41%), and 0.05% (0.02, 0.09%), respectively. As for the single lag days, the cumulative lag days ranged from lag01 to lag 05. PM_2.5_, PM_10_, SO_2_, and NO_2_ had the greatest cumulative effects on lag02. For O_3_, the cumulative effects on lag03 were the largest.

**Figure 3:**
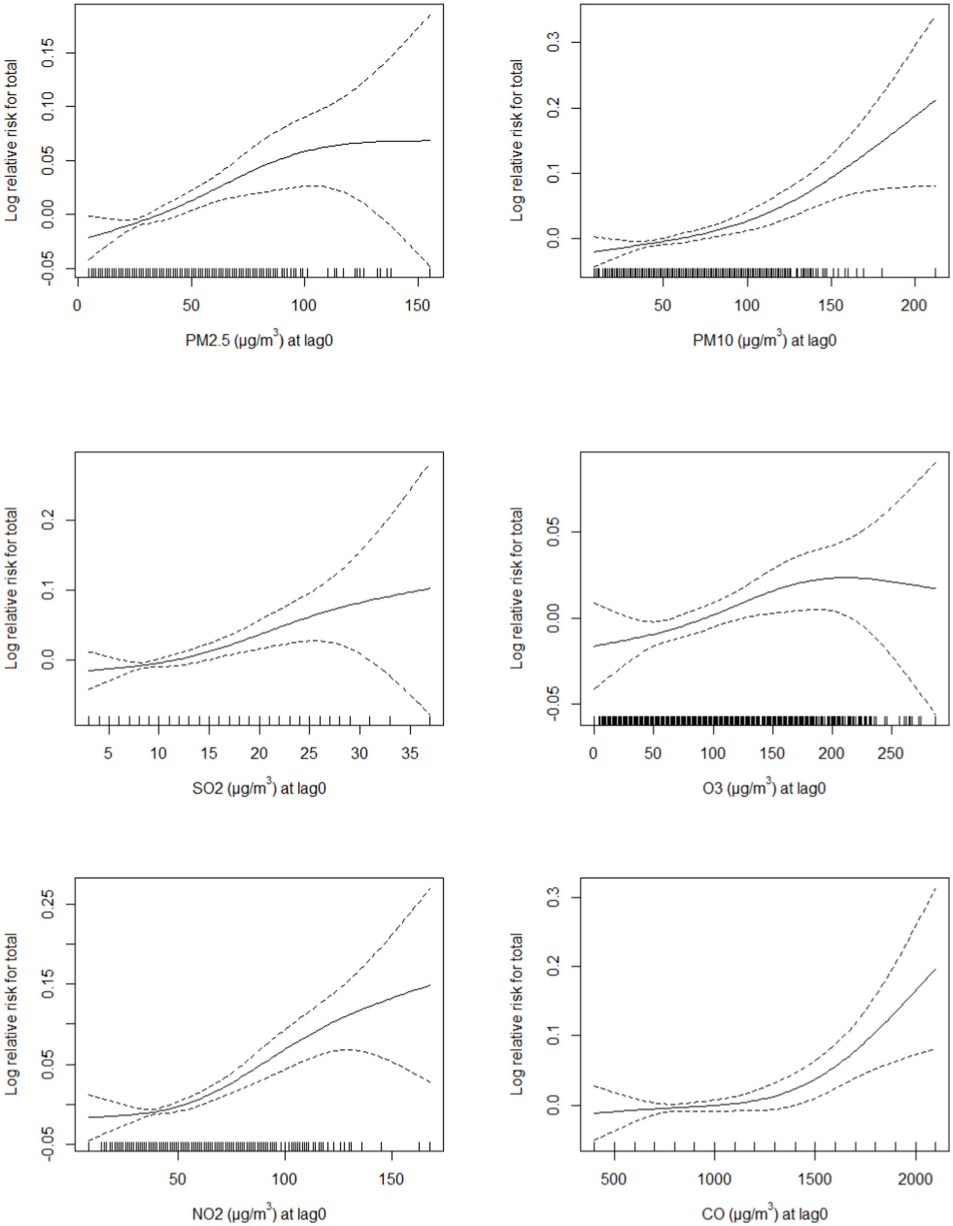
Exposure–response curves of different air pollutants and mortality under a single air pollutant model. The black line represents the average relative risk, and the dashed line represents the 95% confidence interval of the risk estimate. PM_2.5_, fine particulate matter; PM_10_, inhalable particles; SO_2_, sulfur dioxide; O_3_, ozone; NO_2_, nitric dioxide; CO, carbon monoxide.

The exposure–response curves of the associations between air pollution concentrations and mortality are shown in Figure 3. The exposure–response relationships between air pollutants and the risk of mortality were obviously positive. In the statistically significant exposure range, the six ambient air pollution curves showed a significant positive correlation. The exposure–response curves of PM_10_, NO_2_, and CO increased sharply at concentrations >100 μg/m^3^, > 50 μg/m^3^, and >1,400 μg/m^3^, respectively. Moreover, the exposure–response curves of PM_2.5_ and O_3_ were approximately S-shaped, increasing slowly at concentrations from 100 to 200 μg/m^3^ and then plateauing. The SO_2_ exposure–response curves showed an increasing trend from 15 to 35 μg/m^3^.

**Figure 4:**
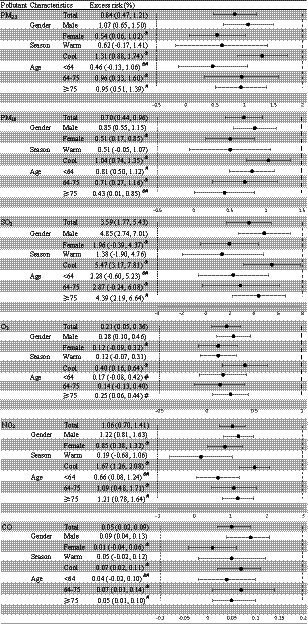
Excess risk (%) and 95% confidence intervals (CIs) for mortality per 10-μg/m^3^ increase in the concentrations of six ambient air pollutants, stratified by sex, age, and season. The warm period was from May 1 to September 30, and the cool period was from October 1 to April 30. Statistically significant estimates are highlighted in bold. ^&^, ^#^Statistically significant between-group differences.

Figure 4 shows the estimated ERs of mortality with 95% CIs, stratified by sex, season, and age. In the stratified analysis, the six ambient air pollutants showed significant differences according to sex, season, and age. There were statistically significant differences in the relationships between ambient air pollutant concentrations and mortality by sex. For all six types of ambient air pollutants, the association effects between mortality and ambient air pollutant concentrations were greater among male than female individuals. In comparison, there were significant relationships between SO_2_, O_3_, and CO concentrations and mortality among male but not female individuals. There also were significant relationships between mortality and all six ambient air pollutant concentrations in male individuals. The age-stratified analysis showed significant differences between people aged <64 and those aged ≥64 years. There were significant associations between mortality and all six ambient air pollutants among people aged ≥75 years. Moreover, the PM_10_ and NO_2_ concentrations were significantly associated with mortality among people aged <64, 64–75, and ≥75 years. The stratified analysis by season showed significant differences in the association between ambient air pollutants and the risk of mortality. Mortality associated with the six air pollutants was more significant in the cool season. The relationship between air pollutant concentrations and the number of deaths was significantly different in the cool season but not the warm season.

**Figure 5:**
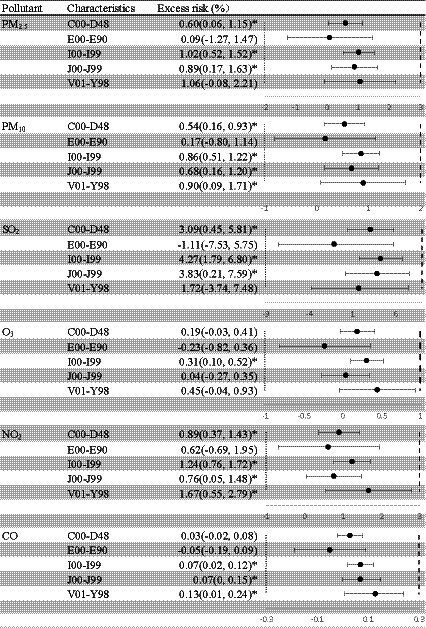
Excess risk (%) and 95% confidence intervals (CIs) for mortality per 10-μg/m^3^ increase in the concentrations of air pollutants, stratified by cause of death. C00–D48, neoplasms; E00–E90, endocrine, nutritional, and metabolic diseases; I00–I99, diseases of the circulatory system; J00–J99, diseases of the respiratory system; V01–Y98, external causes of morbidity and mortality. Statistically significant estimates are highlighted in bold. PM_2.5_, fine particulate matter; PM_10_, inhalable particles; SO_2_, sulfur dioxide; O_3_, ozone; NO_2_, nitric dioxide; CO, carbon monoxide. The “^*^” means *p* < 0.05.

The disease stratification analysis presented in Figure 5 shows that there were significant effects of the six air pollutants on the risk of circulatory system diseases. However, the six ambient pollutants had no significant effects on endocrine, nutritional, or metabolic diseases. The relationships between PM_10_ and NO_2_ concentrations and diseases other than endocrine, nutritional, and metabolic diseases were significant. Specifically, only the association between O_3_ concentration and circulatory diseases was statistically significant. For PM_2.5_ and SO_2_, the associations were more significant for neoplasms, circulatory diseases, and respiratory diseases than for other diseases.

**Figure 6:**
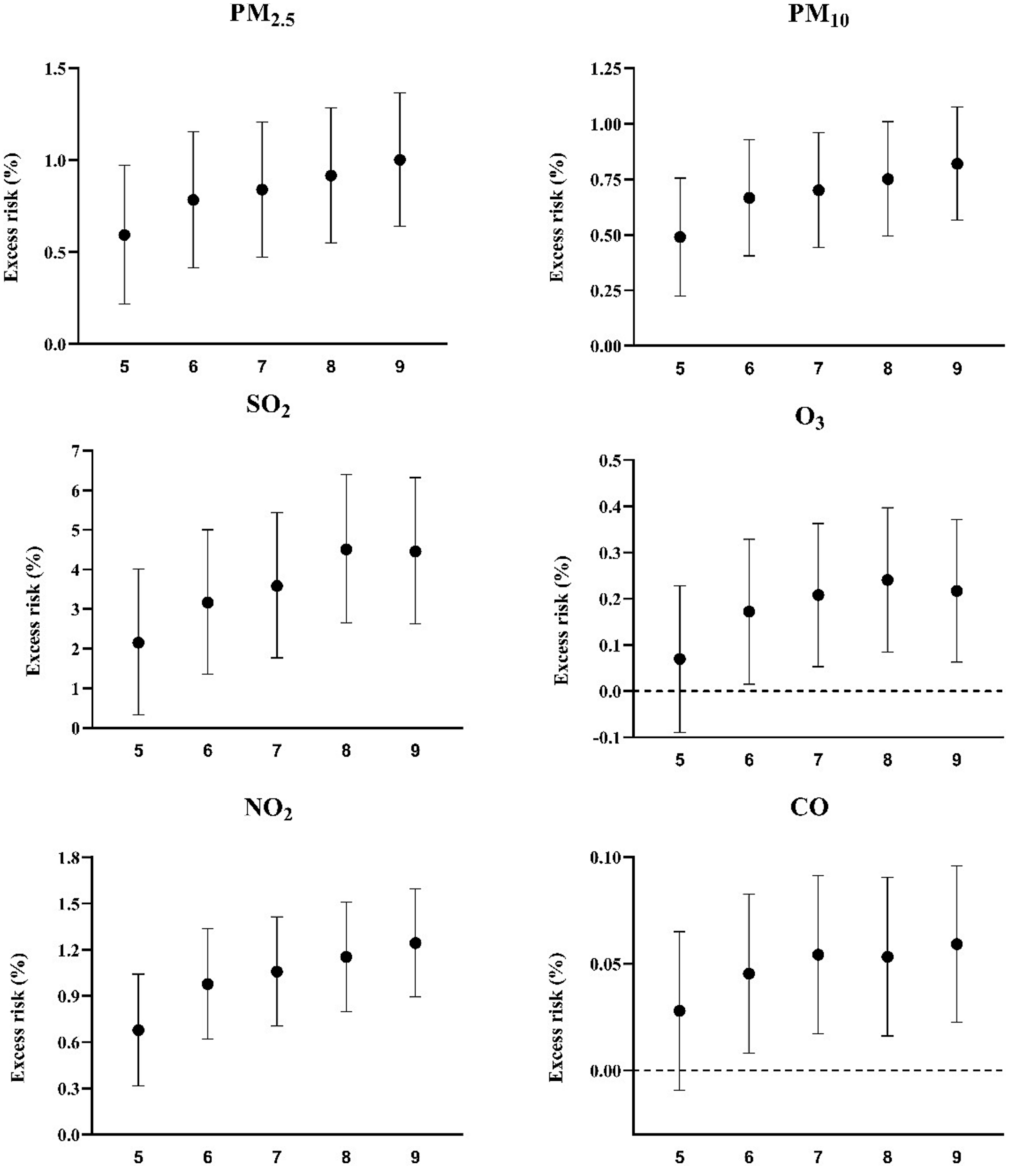
Excess risk (%) and 95% confidence intervals for mortality per 10-μg/m^3^ increase in pollutant concentrations on the current day using different degrees of freedom per year. PM_2.5_, fine particulate matter; PM_10_, inhalable particles; SO_2_, sulfur dioxide; O_3_, ozone; NO_2_, nitric dioxide; CO, carbon monoxide.

In the sensitivity analysis, when the df for time were transformed from 5 to 9, the results did not change substantially, except for CO and O_3_ (Figure 6).

Table 2 shows the relationships between the concentrations of the six air pollutants and mortality in the two-pollutant models, which were also used for sensitivity analysis. Co-pollutants with a Spearman’s correlation coefficient <0.7 were added to the two-pollutant models. The associations between SO_2_, O_3_, and CO concentration and mortality were likely affected by other air pollutants, but PM_2.5_, PM_10_, and NO_2_ may have played more independent roles in the mortality risk. The associations between PM_2.5_, PM_10_, and NO_2_ concentrations and the risk of mortality remained robust in the co-pollutant models. After adjusting for the PM_2.5_, PM_10_, SO_2_, and NO_2_ concentrations, the effects of O_3_ decreased and became non-significant. After adjusting for the PM_2.5_, PM_10_, and NO_2_ concentrations, the effects of SO_2_ were less non-significant. Taken together, these results suggested that PM_2.5_, PM_10_, and NO_2_ may play more essential individual roles in mortality risk.

**Table 2:** Two-pollutant models of excess risk (%) and 95% confidence intervals for mortality.

Two-pollutant models		Estimates, % (95% CI)
PM_2.5_	−	0.84 (0.47, 1.21)^*^
	+ SO_2_	0.65 (0.13, 1.17)^*^
	+ O_3_	0.79 (0.37, 1.21)^*^
	+ CO	0.85 (0.37, 1.33)^*^
PM_10_	−	0.70 (0.44, 0.96)^*^
	+ SO_2_	0.71 (0.32, 1.09)^*^
	+ O_3_	0.71 (0.41, 1.00)^*^
	+ CO	0.77 (0.44, 1.11)^*^
SO_2_	−	3.59 (1.77, 5.43)^*^
	+ PM_2.5_	1.31 (−1.19, 3.87)
	+ PM_10_	−0.07 (−2.66, 2.59)
	+ O_3_	3.11 (1.14, 5.11)^*^
	+ NO_2_	−0.41 (−2.86, 2.11)
	+ CO	3.00 (0.97, 5.07)^*^
O_3_	−	0.21 (0.05, 0.36)^*^
	+ PM_2.5_	0.05 (−0.13, 0.22)
	+ PM_10_	0 (−0.18, 0.17)
	+ SO_2_	0.10 (−0.07, 0.27)
	+ NO_2_	0 (−0.17, 0.18)
	+ CO	0.16 (0, 0.32)^*^
NO_2_	−	1.06 (0.70, 1.41)^*^
	+ SO_2_	1.12 (0.61, 1.62)^*^
	+ O_3_	1.05 (0.66, 1.45)^*^
	+ CO	1.12 (0.69, 1.56)^*^
CO	−	0.05 (0.02, 0.09)^*^
	+ PM_2.5_	0 (−0.05, 0.05)
	+ PM_10_	−0.02 (−0.06, 0.03)
	+ SO_2_	0.03 (−0.02, 0.07)
	+ O_3_	0.04 (0.01, 0.08)^*^
	+ NO_2_	−0.01 (−0.06, 0.03)

*P< 0.05. CI, confidence interval; PM2.5, fine particulate matter; PM10, inhalable particles; SO2, sulfur dioxide; O3, ozone; NO2, nitric dioxide; CO, carbon monoxide.

## 4 Discussion

We designed a time-series study based on daily mortality data and air pollutant concentrations from 2015 to 2019, and observed significant relationships between the concentrations of six air pollutants and mortality. In the statistically significant exposure range, the exposure–response curves of the detected ambient air pollutants were significantly and positively correlated with mortality risk. According to the analysis stratified by sex, the associations between mortality and the six ambient air pollutant concentrations were stronger among male than among female individuals. Furthermore, the effects of these six pollutants on mortality were more obvious during the cold season than the warm season. In the disease stratification analysis, we found statistically significant associations between the different air pollutants and all diseases studied except endocrine, nutritional, and metabolic diseases. However, the two-pollutant model showed that PM_2.5_, PM_10_, and NO_2_ may play more essential roles individually than the other air pollutants in determining mortality risk.

In the time-series analysis, PM_2.5_, PM_10_, SO_2_, O_3_, NO_2_, and CO had a greater impact on the risk of mortality in male than in female individuals, which is consistent with the findings of a previous study (27). We speculate that this may be related to greater lung ventilation in men. It is also possible that occupational factors contribute to this result, as men are more likely to perform outdoor work and thus may be exposed to higher concentrations of air pollutants for a longer time (28). Moreover, men may have lower antioxidants levels and weaker ability to detect exogenous poisons and neutralize them, making them more vulnerable to air pollutants (29). However, many studies have found that air pollution has a greater effect on women than men (30). This contradiction may be due to differences in study design, sample selection, or the model protocols used in different studies. The associations between the six air pollutant concentrations and mortality were stronger in the cold season than the warm season. This seasonal change may be caused by changes in the atmospheric boundary layer (17). Moreover, people spend most of their time indoors in the warm season due to the rain and hot weather (31).

The stratified analysis showed that all six environmental pollutants had significant effects on circulatory system diseases but no significant effects on endocrine, nutritional, and metabolic diseases. In addition, PM_2.5_, PM_10_, SO_2_, and NO_2_ had stronger associations with neoplasms and respiratory and circulatory diseases than the other two. Many studies have demonstrated that long-term exposure to air pollutants, especially PM_2.5_ (32, 33), is associated with respiratory diseases. As it comprises small inhaled particulate matter, PM_2.5_ can penetrate the line of defense of the respiratory system, act on alveolar epithelial cells, and promote the release of pro-inflammatory mediators and vasoactive factors (34). It can interact with receptors on nerves lung cells, causing an imbalance in the autonomic nervous system, which can cause heart rhythm disturbances (35). Circulatory system diseases are also clearly related to air pollution (35, 36). PM_2.5_ promotes the entry of microorganisms, such as viruses, into the human body through a variety of mechanisms, because the size of these particles makes it easier for them to enter the systemic circulation (37). Furthermore, studies have shown that long-term exposure to air pollution is associated with vascular inflammation and atherosclerosis (35, 38, 39). Other studies have shown a link between long-term exposure to PM_2.5_, PM_10_, and NO_2_ and heart failure (40, 41). NO_2_ can directly cause damage to the respiratory system and penetrate the respiratory membrane to damage the cardiovascular system (34, 42). It also enhances the Th1/Th17 immune response and promotes the release of proinflammatory factors, which may promote the development of endobronchial spasm (36, 43, 44). PM_2.5_ can reach the blood through the alveoli, inducing the production of reactive oxygen species and reactive nitrogen species and stimulating lung inflammation (45). As a respiratory irritant, SO_2_ inhibits bronchial cilia from clearing mucus. SO_2_ forms bisulfite after deep penetration into the respiratory system, and both SO_2_ and bisulfite stimulate sensory receptors, resulting in bronchial contraction, which causes respiratory diseases (34, 46, 47). Our research found that the adverse effects of SO_2_ disappeared or reversed in the co-pollutant model, which was consistent with previous study (1). The results suggest that SO_2_ is easily affected by other pollutants and further studies are needed. Some studies have reported that PM_2.5_ acts on cells with carcinogenic mutations in healthy lung tissue, causing them to become cancerous (48). Epidermal growth factor receptor-driven carcinogenesis by PM_2.5_-induced mutagenesis is a common cause of lung cancer in nonsmokers and light smokers (42). After being engulfed by macrophages, air pollutants enter the lung tissue, prompting macrophages to release interleukin-1β, which leads to changes in the morphology of type II alveolar epithelial cells (49). Long-term exposure to PM_2.5_ is associated with increased lung cancer risk and mortality (50). Our study addresses the issue of the paucity of studies examining the association between air pollutants other than PM_2.5_, such as NO_2_, and cancer, respiratory, and cardiovascular diseases and provides the direction for our future work.

However, our study has some limitations. First, the air pollutant concentrations and meteorological data analyzed in this study were obtained from fixed-site monitors, rather than from individual monitors. Moreover, the pollutant and meteorological data were obtained only from monitoring stations, and the concentrations obtained may be different from the actual exposure concentrations of the population. Thus, there may have been exposure measurement errors. Second, the causes of death included in the analysis were not sufficiently diverse. Due to the small sample size for other mortality classifications, our study only included a stratified analysis of five causes of death, which may not be representative. Third, we did not include all air pollutants in the scope of the study, but we only focused on six major air pollutants. This may have led to an inadequate understanding of how pollutants interact with each other. Fourth, some unmeasured confounders may have affected the results.

## 5 Conclusion

This time-series analysis showed that ambient air pollutants significantly increase the risk of daily all-cause mortality in Guangzhou. In particular, the individual effects of PM_2.5_, PM_10_, and NO_2_ on the risk of mortality were stronger than the effects of SO_2_, CO, and O_3_. All six air pollutants had a more significant effect on mortality risk in men than in women. People aged ≥64 years were more likely to be affected by atmospheric pollutants than those aged <64. Moreover, the relationship between the six environmental pollutants and mortality was more significant in the cold season than in the warm season. The effects of PM_2.5_, PM_10_, SO_2_, and NO_2_ were more obvious on cancer and respiratory and circulatory diseases than on the other diseases analyzed. It was noteworthy that all six pollutants had effects on circulatory system diseases. These results suggest that efforts to reduce air pollution would be beneficial in reducing the risk of mortality in Guangzhou.

## Data availability statement

The original contributions presented in the study are included in the article/supplementary material, further inquiries can be directed to the corresponding author.

## Ethics statement

The studies involving humans were approved by the Research Ethics Committee of Guangzhou Medical University. The studies were conducted in accordance with the local legislation and institutional requirements. Written informed consent for participation was not required from the participants or the participants’ legal guardians/next of kin in accordance with the national legislation and institutional requirements.

## Author contributions

YC: Conceptualization, Data curation, Formal analysis, Methodology, Writing – original draft. SC: Conceptualization, Data curation, Methodology, Writing – review & editing. LZ: Data curation, Software, Writing – original draft. WK: Formal analysis, Methodology, Software, Writing – original draft. GL: Investigation, Resources, Validation, Writing – review & editing. QY: Funding acquisition, Project administration, Resources, Supervision, Writing – review & editing.

## Footnotes

^1^https://air.cnemc.cn:18007/

## References

[1] Lu M Ding S Wang J Liu Y An Z Li J. Acute effect of ambient air pollution on hospital outpatient cases of chronic sinusitis in Xinxiang, China. Ecotoxicol Environ Saf. (2020) 202:110923. doi: 10.1016/j.ecoenv.2020.110923, PMID: 32800210

[2] Zhou MG Wang H Zeng X Yin P Zhu J Chen W. Mortality, morbidity, and risk factors in China and its provinces, 1990-2017: a systematic analysis for the global burden of disease study 2017. Lancet. (2019) 394:1145–58. doi: 10.1016/S0140-6736(19)30427-1, PMID: 31248666 PMC6891889

[3] Hadley MB Vedanthan R Fuster V. Air pollution and cardiovascular disease: a window of opportunity. Nat Rev Cardiol. (2018) 15:193–4. doi: 10.1038/nrcardio.2017.207, PMID: 29297510 PMC6070296

[4] Luo L Zhang Y Jiang J Luan H Yu C Nan P. Short-term effects of ambient air pollution on hospitalization for respiratory disease in Taiyuan, China: a time-series analysis. Int J Environ Res Public Health. (2018) 15:2160. doi: 10.3390/ijerph15102160 30275384 PMC6210308

[5] Liu C Cai J Chen R Sera F Guo Y Tong S. Coarse particulate air pollution and daily mortality: a global study in 205 cities. Am J Respir Crit Care Med. (2022) 206:999–1007. doi: 10.1164/rccm.202111-2657OC 35671471

[6] Su C Breitner S Schneider A Liu L Franck U Peters A. Short-term effects of fine particulate air pollution on cardiovascular hospital emergency room visits: a time-series study in Beijing, China. Int Arch Occup Environ Health. (2015) 89:641–57. doi: 10.1007/s00420-015-1102-6, PMID: 26547916

[7] Stafoggia M Oftedal B Chen J Rodopoulou S Renzi M Atkinson RW. Long-term exposure to low ambient air pollution concentrations and mortality among 28 million people: results from seven large European cohorts within the ELAPSE project. Lancet Planet Health. (2022) 6:E9–E18. doi: 10.1016/S2542-5196(21)00277-1, PMID: 34998464

[8] Khaltaev N Axelrod S. Chronic respiratory diseases global mortality trends, treatment guidelines, life style modifications, and air pollution: preliminary analysis. J Thorac Dis. (2019) 11:2643–55. doi: 10.21037/jtd.2019.06.08, PMID: 31372301 PMC6626823

[9] Wang M Zhou T Song Q Ma H Hu Y Heianza Y. Ambient air pollution, healthy diet and vegetable intakes, and mortality: a prospective UK biobank study. Int J Epidemiol. (2022) 51:1243–53. doi: 10.1093/ije/dyac022, PMID: 35179602 PMC9365625

[10] Zhao S Liu S Hou X Sun Y Beazley R. Air pollution and cause-specific mortality: a comparative study of urban and rural areas in China. Chemosphere. (2021) 262:127884. doi: 10.1016/j.chemosphere.2020.127884, PMID: 33182102

[11] Masroor K Shamsipour M Mehrdad R Fanaei F Aghaei M Yunesian M. Exposure to ambient gaseous air pollutants and adult lung function: a systematic review. Rev Environ Health. (2023) 38:137–50. doi: 10.1515/reveh-2021-0135, PMID: 34957731

[12] Wang N Mengersen K Tong S Kimlin M Zhou M Wang L. Short-term association between ambient air pollution and lung cancer mortality. Environ Res. (2019) 179:108748. doi: 10.1016/j.envres.2019.108748, PMID: 31561053

[13] Zeng X Jin S Chen X Qiu Y. Association between ambient air pollution and pregnancy outcomes in patients undergoing in vitro fertilization in Chengdu, China: a retrospective study. Environ Res. (2020) 184:109304. doi: 10.1016/j.envres.2020.109304, PMID: 32192988

[14] Wang H Tian C Wang W Luo X. Temporal cross-correlations between ambient air pollutants and seasonality of tuberculosis: a time-series analysis. Int J Environ Res Public Health. (2019) 16:1585. doi: 10.3390/ijerph16091585 31064146 PMC6540206

[15] Zheng Y Chen S Chen Y Li J Xu B Shi T. Association between PM2.5-bound metals and pediatric respiratory health in Guangzhou: An ecological study investigating source, health risk, and effect. Front Public Health. (2023) 11:1137933. doi: 10.3389/fpubh.2023.1137933 36969623 PMC10033947

[16] Wang HL Sun J Qian ZM Gong YQ Zhong JB Yang RD. Association between air pollution and atopic dermatitis in Guangzhou, China: modification by age and season*. Br J Dermatol. (2021) 184:1068–76. doi: 10.1111/bjd.19645, PMID: 33131069

[17] Zhang H Wang Y Hu J Ying Q Hu XM. Relationships between meteorological parameters and criteria air pollutants in three megacities in China. Environ Res. (2015) 140:242–54. doi: 10.1016/j.envres.2015.04.004 25880606

[18] Leong M Karr CJ Shah SI Brumberg HL. Before the first breath: why ambient air pollution and climate change should matter to neonatal-perinatal providers. J Perinatol. (2022) 43:1059–66. doi: 10.1038/s41372-022-01479-2, PMID: 36038659 PMC9421104

[19] Adamkiewicz G Liddie J Gaffin JM. The respiratory risks of ambient/outdoor air pollution. Clin Chest Med. (2020) 41:809–24. doi: 10.1016/j.ccm.2020.08.013, PMID: 33153697 PMC7665094

[20] Wu K Ho HC Su H Huang C Zheng H Zhang W. A systematic review and meta-analysis of intraday effects of ambient air pollution and temperature on cardiorespiratory morbidities: first few hours of exposure matters to life. EBioMedicine. (2022) 86:104327. doi: 10.1016/j.ebiom.2022.104327, PMID: 36323182 PMC9626385

[21] Liu W Zhang Q Liu W Qiu C. Association between air pollution exposure and gestational diabetes mellitus in pregnant women: a retrospective cohort study. Environ Sci Pollut Res. (2022) 30:2891–903. doi: 10.1007/s11356-022-22379-0, PMID: 35941503

[22] Zhao QG Liang Z Tao S Zhu J du Y. Effects of air pollution on neonatal prematurity in Guangzhou of China: a time-series study. Environ Health. (2011) 10:2. doi: 10.1186/1476-069X-10-2, PMID: 21214958 PMC3024279

[23] Zhang S Lv J Meng R Yang Y Acharya BK Sun X. The association between ambient air pollution control and stroke mortality during the 2010 Asian games in Guangzhou, China. Atmos Environ. (2019) 217:116965. doi: 10.1016/j.atmosenv.2019.116965

[24] Lu J Wu K Ma X Wei J Yuan Z Huang Z. Short-term effects of ambient particulate matter (PM1, PM2.5 and PM10) on influenza-like illness in Guangzhou, China. Int J Hyg Environ Health. (2023) 247:114074. doi: 10.1016/j.ijheh.2022.114074 36436470

[25] Li W Pei L Li A Luo K Cao Y Li R. Spatial variation in the effects of air pollution on cardiovascular mortality in Beijing, China. Environ Sci Pollut Res. (2018) 26:2501–11. doi: 10.1007/s11356-018-3725-0 30471063

[26] Chen S Xu B Shi T Yang Q. Short-term effect of ambient air pollution on outpatient visits for children in Guangzhou, China. Front Public Health. (2023) 11:1058368. doi: 10.3389/fpubh.2023.1058368, PMID: 36741946 PMC9895100

[27] Zeng X-W Qian Z(M) Vaughn MG Nelson EJ Dharmage SC Bowatte G. Positive association between short-term ambient air pollution exposure and children blood pressure in China–result from the seven northeast cities (SNEC) study. Environ Pollut. (2017) 224:698–705. doi: 10.1016/j.envpol.2017.02.054 28259583

[28] Wang J Lu M An Z Jiang J Li J Wang Y. Associations between air pollution and outpatient visits for allergic rhinitis in Xinxiang, China. Environ Sci Pollut Res. (2020) 27:23565–74. doi: 10.1007/s11356-020-08709-0, PMID: 32291645

[29] Wang S Li Y Niu A Liu Y Su L Song W. The impact of outdoor air pollutants on outpatient visits for respiratory diseases during 2012–2016 in Jinan, China. Respir Res. (2018) 19:246. doi: 10.1186/s12931-018-0958-x 30541548 PMC6292059

[30] Mo S Wang Y Peng M Wang Q Zheng H Zhan Y. Sex disparity in cognitive aging related to later-life exposure to ambient air pollution. Sci Total Environ. (2023) 886:163980. doi: 10.1016/j.scitotenv.2023.163980 37150467

[31] Yang H Yan C Li M Zhao L Long Z Fan Y. Short term effects of air pollutants on hospital admissions for respiratory diseases among children: a multi-city time-series study in China. Int J Hyg Environ Health. (2021) 231:113638. doi: 10.1016/j.ijheh.2020.113638, PMID: 33080524

[32] Zosky GR. Air pollution and respiratory health—do we really need more evidence? Respirology. (2023) 28:513–4. doi: 10.1111/resp.14490, PMID: 36918344

[33] Cong X Zhang J Sun R Pu Y. Short-term ambient particulate air pollution exposure, microRNAs, blood pressure and lung function. Environ Pollut. (2022) 292:118387. doi: 10.1016/j.envpol.2021.118387, PMID: 34673158

[34] Manisalidis I Stavropoulou E Stavropoulos A Bezirtzoglou E. Environmental and health impacts of air pollution: a review. Front Public Health. (2020) 8:8. doi: 10.3389/fpubh.2020.00014 32154200 PMC7044178

[35] Hayes RB Lim C Zhang Y Cromar K Shao Y Reynolds HR. PM2.5 air pollution and cause-specific cardiovascular disease mortality. Int J Epidemiol. (2020) 49:25–35. doi: 10.1093/ije/dyz114, PMID: 31289812 PMC7124502

[36] Rajagopalan S Al-Kindi SG Brook RD. Air pollution and cardiovascular disease: JACC state-of-the-art review. J Am Coll Cardiol. (2018) 72:2054–70. doi: 10.1016/j.jacc.2018.07.099 30336830

[37] Yang B-Y Guo Y Markevych I Qian Z(M) Bloom MS Heinrich J. Association of Long-term Exposure to ambient air pollutants with risk factors for cardiovascular disease in China. JAMA Netw Open. (2019) 2:e190318. doi: 10.1001/jamanetworkopen.2019.0318, PMID: 30848806 PMC6484675

[38] Newby DE Mannucci PM Tell GS Baccarelli AA Brook RD Donaldson K. Expert position paper on air pollution and cardiovascular disease. Eur Heart J. (2015) 36:83–93. doi: 10.1093/eurheartj/ehu458, PMID: 25492627 PMC6279152

[39] Nemmar A Hoylaerts MF Nemery B. Effects of particulate air pollution on hemostasis. Clin Occup Environ Med. (2006) 5:865–81. doi: 10.1016/j.coem.2006.07.007, PMID: 17110297

[40] Wang M Zhou T Song Y Li X Ma H Hu Y. Joint exposure to various ambient air pollutants and incident heart failure: a prospective analysis in UK biobank. Eur Heart J. (2021) 42:1582–91. doi: 10.1093/eurheartj/ehaa1031, PMID: 33527989 PMC8060055

[41] Bhatnagar A. Cardiovascular effects of particulate air pollution. Annu Rev Med. (2022) 73:393–406. doi: 10.1146/annurev-med-042220-011549, PMID: 34644154 PMC10132287

[42] Christiani DC. Ambient air pollution and lung cancer: nature and nurture. Am J Respir Crit Care Med. (2021) 204:752–3. doi: 10.1164/rccm.202107-1576ED 34370960 PMC8528534

[43] Nishida C Yatera K. The impact of ambient environmental and occupational pollution on respiratory diseases. Int J Environ Res Public Health. (2022) 19:2788. doi: 10.3390/ijerph19052788, PMID: 35270479 PMC8910713

[44] Brook RD Rajagopalan S Pope CA Brook JR Bhatnagar A Diez-Roux AV. Particulate matter air pollution and cardiovascular disease: An update to the scientific statement from the American Heart Association. Circulation. (2010) 121:2331–78. doi: 10.1161/CIR.0b013e3181dbece1 20458016

[45] Thangavel P Park D Lee Y-C. Recent insights into particulate matter (PM2.5)-mediated toxicity in humans: an overview. Int J Environ Res Public Health. (2022) 19:7511. doi: 10.3390/ijerph19127511, PMID: 35742761 PMC9223652

[46] Danesh Yazdi M Wei Y di Q Requia WJ Shi L Sabath MB. The effect of long-term exposure to air pollution and seasonal temperature on hospital admissions with cardiovascular and respiratory disease in the United States: a difference-in-differences analysis. Sci Total Environ. (2022) 843:156855. doi: 10.1016/j.scitotenv.2022.156855, PMID: 35750164 PMC10007814

[47] Wang X Chen L Cai M Tian F Zou H Qian Z(M). Air pollution associated with incidence and progression trajectory of chronic lung diseases: a population-based cohort study. Thorax. (2023) 78:698–705. doi: 10.1136/thorax-2022-219489, PMID: 36732083

[48] Wu X Zhu B Zhou J Bi Y Xu S Zhou B. The epidemiological trends in the burden of lung cancer attributable to PM2.5 exposure in China. BMC Public Health. (2021) 21:737. doi: 10.1186/s12889-021-10765-1, PMID: 33858412 PMC8051098

[49] Sierra-Vargas MP Montero-Vargas JM Debray-García Y Vizuet-de-Rueda JC Loaeza-Román A Terán LM. Oxidative stress and air pollution: its impact on chronic respiratory diseases. Int J Mol Sci. (2023) 24:853. doi: 10.3390/ijms24010853, PMID: 36614301 PMC9821141

[50] Hill W Lim EL Weeden CE Lee C Augustine M Chen K. Lung adenocarcinoma promotion by air pollutants. Nature. (2023) 616:159–67. doi: 10.1038/s41586-023-05874-3, PMID: 37020004 PMC7614604

